# Bacteria and Carcinogenesis and the Management of Cancer: A Narrative Review

**DOI:** 10.3390/pathogens14050509

**Published:** 2025-05-21

**Authors:** Paulina Plewa, Kajetan Kiełbowski, Oliwia Mentel, Karolina Figiel, Estera Bakinowska, Rafał Becht, Bolesław Banach, Andrzej Pawlik

**Affiliations:** 1 Department of Physiology, Pomeranian Medical University, 70-111 Szczecin, Poland; paulina.plewa@op.pl (P.P.);; 2 Department of Clinical Oncology, Chemotherapy and Cancer Immunotherapy, Pomeranian Medical University, 71-252 Szczecin, Poland

**Keywords:** cancer, bacteria, microbiome, tumor microenvironment, immunotherapy

## Abstract

There is a widely known relationship between certain microbes and cancer progression. For instance, *Helicobacter pylori* is associated with the occurrence of gastric cancer, while HPV is associated with cervical and head and neck cancers. Recent studies have uncovered novel and important associations between bacterial presence and tumor formation and treatment response. Apart from the influence of the intestinal microbiome on cancer, the local activity of bacteria affects disease properties as well. Bacteria can localize within tumors in less vascularized niches. Their presence mediates the activity of signaling pathways, which contribute to tumorigenesis. Furthermore, they affect the composition of the tumor microenvironment, a highly complex structure composed of immunoregulatory cells and secreted inflammatory mediators. Recently, researchers have analyzed the properties of bacteria to develop novel anticancer strategies. The aim of this review is to discuss the latest findings regarding the relationships between bacteria and cancer and the properties of bacteria that could be used to kill cancer cells.

## 1. Introduction

Cancer research is one of the most advanced fields of medicine. Treatment guidelines and local regulations change frequently, thus offering patients access to novel and innovative treatment approaches. Translational medicine, the concept of research commonly referred to as “from bench to bedside”, plays a significant role in cancer studies, as preclinical findings drive clinical trial designs. Over the years, researchers revealed numerous mechanisms involved in the pathogenesis of cancer. Genetic and environmental factors are frequently distinguished. Epigenetics represents another key branch contributing to tumor occurrence. Additionally, it is widely known that microbes are strongly linked to the mechanisms leading to cancer development. Viruses like human papilloma virus (HPV), hepatitis B and C viruses (HBV, HCV), or Epstein–Barr virus (EBV) are some of the microbes related to carcinogenesis. Moreover, the relationship between certain bacteria and cancer is also established. Infection with *Helicobacter pylori* is a primary example. Recently, the relationship between bacteria and cancer has been extensively examined. The influence of the gut microbiome or intratumoral bacteria on cancer properties and treatment response is currently being studied. The aim of this review is to discuss the latest findings related to the influence of bacteria on cancer. Furthermore, it will present current concepts regarding the use of bacteria in anticancer therapy.

## 2. Immunoregulatory Properties of Bacteria

### 2.1. Immunoregulatory Properties of Bacterial Metabolites

Bacteria have natural properties associated with stimulating the human immune system, making them an ideal opportunity to fight cancer [1]. This is mainly due to their metabolites, of which only a small portion have been understood. Given the differences in diet between people, there is a significant interpersonal difference in the range and amount of certain metabolites. This is related to the process of metabolism of proteins, fats, and polysaccharides, in which the gut microbiome plays a significant role [2]. Short-chain fatty acids (SCFAs), which include acetate, propionate, and butyrate, are primarily associated with the microbial breakdown of dietary fiber, specifically its fermentation [3]. Their role in immunoregulation is related to mechanisms blocking carcinogenesis, as well as antiproliferative processes. SCFAs, acting as extracellular and intracellular signaling molecules, can function as histone deacetylase inhibitors and ligands of G-protein-coupled receptors (GPCRs). As a result, regulatory T cells (Tregs) proliferate and differentiate, which in turn increases the release of IL-10 and transforming growth factor-β (TGF-β), as well as inhibiting the release of cytokines within macrophages and neutrophils. In addition, it suppresses Th17 differentiation, thus reducing inflammation and carcinogenesis. Butyrate may additionally affect the antitumor response of CD8+ T cells. This is possible due to the control of signaling pathways occurring within dendritic cells (DCs), in particular those related to molecules such as IL-12, IL-27, and IFN-β [2]. SCFAs may also improve the intestinal epithelial barrier as a result of more efficient mucus production, leading to an effect on goblet cells and genes responsible for mucus expression [4].

### 2.2. Direct Role of Bacteria

**Figure 1:**
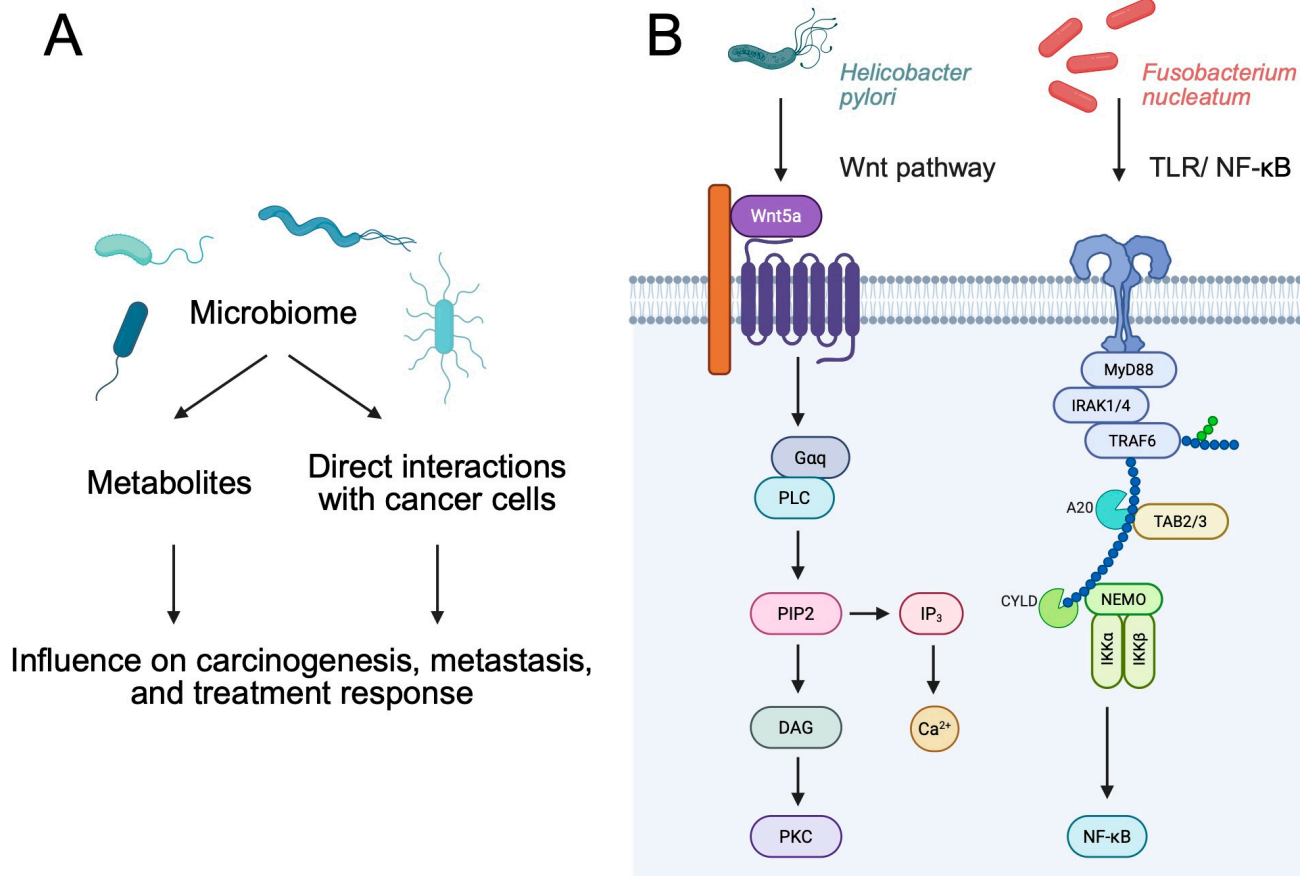
(**A**) Overview of the role of the microbiome in cancer pathophysiology. (**B**) Bacteria can directly promote the activation of signaling pathways that are related to carcinogenesis. For instance, *H. pylori* is associated with the Wnt cascade, while *Fusobacterium necrophorum* is related to TLR/Nf-kB signaling. Created in BioRender. Physiology, D. (2025) https://BioRender.com/mspgl8y.

Many species of bacteria have the ability to block the initiation and proliferation of tumors as a result of the release of various toxins. One of them is an enterotoxin produced by *Clostridium perfringens*, which binds to transmembrane proteins, especially those with claudin-3 and -4, which are found in cancers. As a result of this interaction, cancer cells are blocked [5]. When comparing modified bacteria with unmodified bacteria, it is clear that the former show greater clinical potential. This is mainly related to the increased ability to accumulate within the tumor microenvironment (TME) and stimulate the human immune system, which in turn significantly affects the effectiveness and precision of the treatment. Immunoregulation can occur through processes related to the expression of tumor antigens, but also as a result of immune regulatory factors [6]. *Listeria monocytogenes* and *Salmonella enterica* have a natural ability to present antigens to antigen-presenting cells (APCs). This leads to the activation of mechanisms related to the antitumor response [7]. If an immunoregulatory factor such as α-galactosylceremide is introduced into *L. monocytogenes*, the NK cells can be stimulated, thus enhancing cytotoxicity and the inhibition of metastatic processes [8]. In addition, the introduction of a radioisotope of rhenium into *L. monocytogenes* may contribute significantly to the acquisition of radioactive abilities by bacteria, which would more effectively lead to the removal of cancerous tissue without disturbing healthy tissue [9] (Figure 1).

## 3. Bacteria as Cancer Promoters

Chronic bacterial colonization has been demonstrated to cause a persistent inflammatory state that plays a critical role in carcinogenesis [10,11,12]. The continuous presence of bacteria triggers sustained inflammatory responses that not only inflict direct tissue damage but also foster an immunosuppressive environment, thereby enhancing tumor survival and progression [13]. Persistent infections stimulate the prolonged release of pro-inflammatory cytokines, such as TNF-α, IL-6, IL-8, IL-1β, IL-17F, IL-21, IL-22, and IL-23. Furthermore, they promote the release of reactive nitrogen species and reactive oxygen species (ROS), which cause continuous DNA damage and compromise DNA repair processes, leading to the accumulation of mutations that drive malignant transformation, e.g., in colorectal mucosa [11,12,14,15,16]. Chronic bacterial infections, with prolonged antigen exposure, further contribute to immune evasion by leading to dysregulated immune responses through the continuous recruitment of regulatory immune cells (e.g., cytotoxic T cells and NK cells) and production of immunomodulatory molecules, thereby inhibiting effective antitumor immunity [17]. Furthermore, by releasing immunomodulatory molecules and altering the local cytokine balance, bacteria attract additional suppressive cell types (for example, regulatory T cells, myeloid-derived suppressor cells, and tumor-associated macrophages) and reduce the cytotoxic functions of CD8+ T cells, fostering a tumor-permissive niche that supports cancer progression [13,14].

Direct tumor interactions from bacteria involve their ability to physically engage with host cells, thereby modulating signaling pathways that promote tumorigenesis, invasion, and metastasis [10,12,15]. Certain bacteria secrete virulence factors that disrupt normal cell–cell adhesion and activate oncogenic signaling cascades [12,15]. For instance, bacterial adhesins and secreted toxins bind to cellular receptors. One such example is *H. pylori*, which delivers the E-cadherin-interacting CagA protein to epithelial cells, thereby activating intracellular signaling cascades such as the Wnt/beta-catenin pathway and upregulating oncogenes such as c-Myc and cyclin D1, which in turn promote cell proliferation and survival [12,14,15,16]. Similarly, *Fusobacterium nucleatum* expresses adhesins that bind to epithelial receptors (E-cadherin), thereby triggering intracellular signals that enhance the cellular proliferation and invasion of colorectal cancer (CRC) [18]. It is through these direct interactions that bacteria induce the transformation of normal cells into malignant ones, whilst concurrently establishing a tumor microenvironment that provides further support for cancer progression.

Bacterial infections contribute to oncogenesis by means of an intricate sequence of events that combine direct DNA damage with epigenetic dysregulation [10,19]. In particular, certain bacteria have been demonstrated to cause oxidative stress that directly damages DNA while also inducing epigenetic alterations—such as changes in DNA methylation and histone modifications—that disrupt normal gene regulation [19]. For example, *H. pylori* interferes with DNA repair and silences tumor-suppressing microRNAs [13,19]. Furthermore, the exacerbating effects of bacterial biofilms, such as those produced by *Escherichia coli*, can cause double-strand DNA breaks, leading to mutations and genomic instability while simultaneously triggering epigenetic changes that silence key regulatory genes [14,19]. These genotoxic and epigenetic effects foster a tumor-promoting microenvironment by impairing genomic stability and altering gene expression [12,15]. Furthermore, bacterial pathogens generate reactive oxygen and nitrogen species that not only inflict additional DNA lesions but also contribute to aberrant promoter methylation, further destabilizing the genome [12,17,19]. Collectively, this interplay between bacterial-induced DNA damage, epigenetic reprogramming, and chronic inflammation creates a highly conductive environment for malignant transformation, highlighting potential targets for integrated antimicrobial and epigenetic therapies.

Bacteria that are commensals of the human intestines, but also those that have found their way into the body as a result of infection, represent potential carcinogens [20]. In the case of the gut microbiome, their special role in the process of carcinogenesis may be related to the production of various types of toxins, enzymes, and oncogenic peptides by bacteria. These products of bacterial metabolism can lead to the initiation of inflammation, disorganization of cell cycle surveillance, and dysregulation of cell pathways [21]. In addition, they can promote the initiation of cancer cell proliferation as a result of changes in the immune system, targeting the host cell DNA [22]. It is also known that the TME itself is a habitat for bacteria, indicating a lack of tumor sterility [23,24].

Firstly, chronic inflammation and the activation of signaling pathways can link the presence of certain bacteria to cancer development. *Streprococcus* and *Veilonella* upregulate the PI3K-AKT pathway. As a result of the interaction of *F. nucleatum* with TLR4, NF-κB is stimulated, which significantly promotes inflammation but also oncogenesis. *Bacteroides fragilis* produces a toxin, which affects the breaking of E-cadherin, while *Porphyromonas gingivalis* secretes gingipain, which stimulates the JAK-STAT pathway and receptor tyrosine kinases, influencing the RAS/RAF/MEK cascade and promoting cellular proliferation [6]. Furthermore, *Salmonella typhi* creates a suitable environment for the development of cancer [25]. It produces typhoid toxins and carcinogenic toxins, e.g., nitrosochemicals, which are mainly responsible for the progression of cancer [26].

Several bacteria are strictly related to a specific type of cancer. One of the most well-known relationships is *H. pylori* and gastric cancer (GC) [27,28]. Since 1994, the bacterium has been recognized by the WHO as a carcinogen characterized by a higher index [28]. Statistically, more than 90% of GC is caused by *H. pylori*, while over 50% of people are carriers of the bacteria [29]. It is estimated that 1% of all infections lead to GC formation [30]. *H. pylori* is classified as a G-bacterium, which is characterized by a spital shape. The optimal habitat for living is a space with a neutral pH located between the layer of mucus secreted by the stomach and its epithelium [21,31]. The carcinogenic predisposition of *H. pylori* is mainly related to its virulence factors, which include the following: cagA (cytotoxin-associated gene A), vacA (vacuolating cytotoxin A), and oipA (outer inflammatory protein) [30]. *H. pylori* can be classified into two types, which differ in pathogenicity. Type I additionally has pathogenicity islands of 40 kb, which contain the CagA protein and the VacA toxin [32]. The presence of this additional structure makes this type more pathogenic. In addition, it increases the severity of the disease and, possibly, the risk of GC [28]. In addition, in a recent report, Sharafutdinov demonstrated that a single-nucleotide polymorphism (in the serine protease HtrA) in *H. pylori* promotes mechanisms responsible for promoting the development of GC. Specifically, it enhances DNA double breaks and stimulates inflammation [33].

The best-known virulence factor is CagA, which is associated with the development of gastric adenocarcinoma. It primarily causes the release of IL-8, which consequently contributes to the changes occurring within the stomach epithelium and then to the initiation of defective cell proliferation. Long-term inflammation can contribute to chronic mucositis and carcinogenesis [28,34]. Additionally, CagA upregulates squalene epoxidase (SQLE), an enzyme that participates in cholesterol metabolism and is associated with several malignancies. In GC, SQLE promotes tumor immune evasion, thus making tumor elimination more difficult [35].

It is likely that VacA intensifies the action of urease by affecting its ability to form cell vacuoles, thus facilitating the diffusion of urea in the mucosa tissue towards the stomach lumen. It has a positive effect on the further colonization of *H. pylori* in the acidic environment of the stomach [36,37]. In addition, VacA also affects he apoptosis as a result of the disorganization of mitochondrial function. In addition, it can limit the immune system and block the proliferation of lymphocytes [21]. The role of CagA and VacA acting separately is very well understood, but their interaction is unknown [28]. OipA has been shown to stimulate the secretion of IL-8 in chronic inflammation [38].

Recently published studies further analyze the impact of *H. pylori* on cancer development, highlighting individual molecules that link the progression from gastritis to GC. The bacterium promotes the expression of Hexokinase Domain Containing 1 (HKDC1), which is responsible for altering the metabolism of carbohydrates and lipids in GC. Moreover, it promotes epithelial-to-mesenchymal transition, thus enhancing the progression of gastritis to GC [39]. *H. pylori* also affects the expression of non-coding RNA (ncRNA) molecules [40,41]. They significantly affect gene expression, and their altered expression is one of the highly investigated processes in oncogenesis.

**Figure 2:**
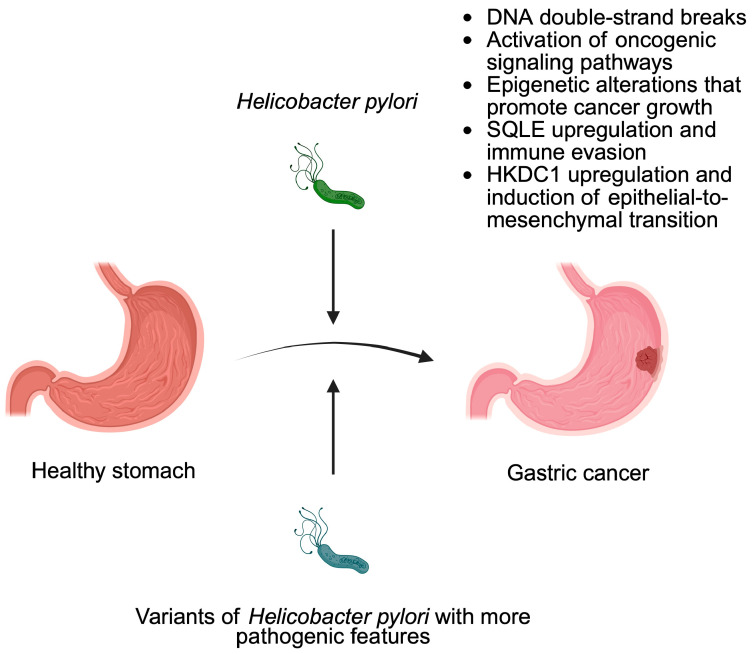
Overview of mechanisms induced by *Helicobacter pylori* that drive the progression of gastric cancer. Created in BioRender. Physiology, D. (2025) https://BioRender.com/2f4x8na.

Importantly, *H. pylori* is associated with microbial dysregulation. Gut dysbiosis is strongly related to the development of diseases, particularly to carcinogenesis. Eradication of *H. pylori* can reverse microbial alterations [42]. Taking into account the relationship between this bacterium and GC, it is important to evaluate clinical data, for instance, if screening for *H. pylori* or its eradication does affect GC mortality. Firstly, non-eradicated *H. pylori* is associated with cancer in general [43]. Secondly, the eradication of bacteria reduces the risk of GC occurrence in healthy individuals. Moreover, the procedure is associated with a lower risk of GC-related mortality. Interestingly, eradication in patients with gastric neoplasia reduces the risks of future GC development [44]. Lee and colleagues [45] reported the results of a large randomized clinical trial that evaluated the usefulness of *H. pylori* stool antigen detection combined with fecal immunochemical testing to prevent GC. The authors observed no impact of stool antigen analysis. Nevertheless, adjustments to the baseline characteristics, length of follow-up, and differences in the screening population led to a reduced risk of GC development (Figure 2).

Furthermore, a significant relationship between *H. pylori* and MALT lymphoma has also been demonstrated [46]. Untreated infections contribute to the initiation of chronic inflammation, leading to the proliferation of T and B cells within the gastric mucosa. It should be borne in mind that the gastric mucosa does not have lymphatic tissue, but as a result of a reaction to chronic inflammation, lymphatic tissue associated with the mucous membrane may form. It is this that leads to malicious changes [47,48]. Current evidence also suggests that *H. pylori* infection is associated with pancreatic cancer [49] and colorectal cancer (CRC) [50].

**Figure 3:**
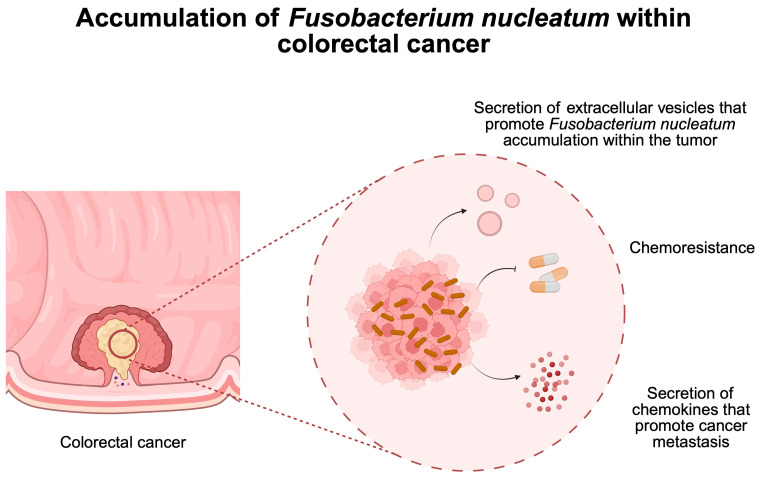
Overview of mechanisms induced by Fusobacterium nucleatum that drive colorectal cancer progression. Created in BioRender. Physiology, D. (2025) https://BioRender.com/0lezl5w.

*F. nucleatum* is another bacterium linked with several malignancies, including CRC [51,52], breast cancer [53], and esophageal cancer [54,55], among others. However, the relationship between *F. nucleatum* and CRC appears to be the most widely investigated. On its surface, it expresses Fad2 (Fusobacterium adhesion), Fap2 (fibroblast activation), and RadD (the radiation gene) but also adhesins, which significantly facilitate the adhesion and attack of epithelial and endothelial cells [56]. The Fad2 protein is necessary for the adhesion and initiation of invasion [28]. The most important pathway affected by Fad2 is the WNT/β-catenin signaling pathway [57]. In addition, the E-cadherin/β-catenin complex is disrupted, resulting in the promotion of epithelial-to-mesenchymal transition [58]. Rad2 acts on CRC cells through the CD147 receptor, resulting in the activation of the PI3K-AKT-NF-κB-MMP signaling pathway and the release of matrix metalloproteinase (MMP). This leads to increased proliferation, migration, and invasion of CRC cells [59,60]. In addition, *F. nucleatum* contributes to epigenetic changes, disorganization in DNA repair, inflammation, and the production of reactive oxygen species and cytokines, which further alter DNA methylation patterns and consequently lead to even greater DNA damage [61,62]. Similarly to *H. pylori*, there seems to be a difference in *F. nucleatum* subtypes that colonize CRC tumors. Zepeda-Rivera et al. [63] observed that a specific clade of *F. nucleatum* colonizes CRC tissue, thus highlighting its specific properties. In a recent discovery by Zheng and collaborators [64], the researchers showed that *F. nucleatum* can secrete extracellular vesicles that will further promote bacterial accumulation within CRC tumors. Thus, recent investigations shed a light on the presence of the bacteria within CRC samples. As a result of their accumulation, CRC tumors progress. Several different mechanisms that explain the role of *F. nucleatum* in CRC pathophysiology have been described. For instance, the bacteria suppress the process of pyroptosis and ferroptosis induced by chemotherapeutic agents [52,65]. Therefore, their greater presence within the CRC can cause chemoresistance. Moreover, through the upregulation of CCL20, *F. nucleatum* promotes the development of CRC metastasis [51]. A greater presence of *F. nucleatum* is also associated with a greater metabolism of the bacteria. Specifically, it produces formate, which was proven to enhance the progression of CRC [66] (Figure 3).

*E. coli* is another bacteria usually present in the colon. However, its contribution has been investigated in several cancers, including cervical cancer [67], CRC [68], and intrahepatic cholangiocarcinoma [69], among others. Strains that have the ability to produce colibactin (*pks* + *E. coli*) have been widely investigated in the context of CRC [70,71]. Colibactin is produced by an enzyme that is encoded at the Pks locus, which contains about 18 genes, including *clbA* (colibactin A) and *clbP* (colibactin P). The action of this toxin is associated with the possibility of DNA damage within enterocytes and significantly facilitates cell proliferation and tumor maturation [28,70]. Furthermore, it is suggested that the presence of *pks* + *E. coli* could be related to driver mutations typical for CRC [72]. In addition, more than 85% of *E. coli* strains with a B2 phenotype that were isolated directly from colorectal adenocarcinoma tissue showed cyclomodulin-positive properties [73].

*Bacteroides fragilis*, although not an appropriate diagnostic factor to determine the prognosis of the disease, is speculated to be related to CRC [74]. *B. fragilis* is classified as a G-bacterium, which, under a microscope, takes on a rod shape. It is relatively anaerobic, and under physiological conditions, is a natural component of the intestinal microflora. The probable link between this bacterium and CRC is related to the production of the BFT toxin mainly by enterotoxic strains of *B. fragilis*. This toxin is encoded by the pathogenicity islands of this bacterium and is associated with the activation of Stat3 within the large intestine, but it also drives the Th17-mediated immune response of mucosa [28,75].

Bacteria are involved in the pathogenesis of cancer in other malignancies as well. Recently, Minot and colleagues [76] showed that a profile of the gut microbiome could suggest the presence of CRC. The colonization of animal models with CRC-related microbes was associated with a significantly greater tumor burden. These important findings show that analysis of the microbiome could potentially lead to the identification of patients with a greater risk of developing CRC. Similar findings were observed in hepatocellular carcinoma, where the fecal transplantation of material from hepatocellular carcinoma (HCC) patients to animal models induced the growth of liver tumors. Mechanistically, further in vivo experiments demonstrated that *Klebsiella Pneumoniae* enhances gut leakage and translocates into the liver, where it promotes carcinogenesis [77].

## 4. Bacteria and Tumor Microenvironment

**Figure 4:**
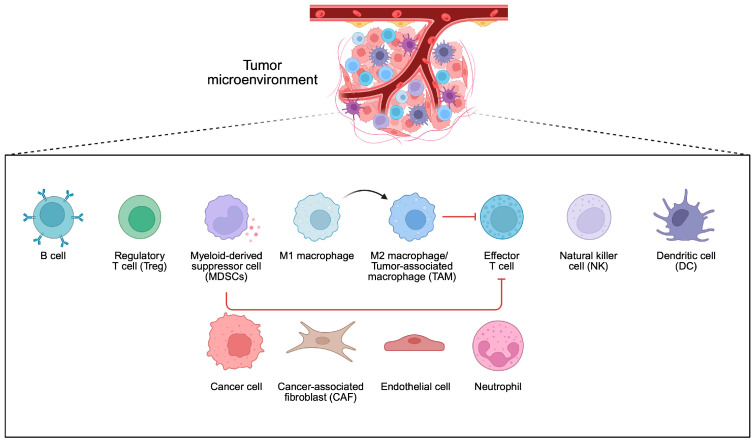
Composition of the tumor microenvironment. Created in BioRender. Physiology, D. (2025) https://BioRender.com/q4dfwa8.

Previous sections have discussed associations between certain bacteria and mechanisms leading to cancer development. Importantly, many of these microbes affect the structure and functionality of the TME. The TME is a complex structure composed of cancer cells, immune cells, mesenchymal cells, and mediators released by these cells (Figure 4). It plays a crucial role in promoting the immunosuppressive conditions surrounding malignant cells, which allow for tumor growth, and is associated with treatment resistance. Through the secretion of cytokines, extracellular vesicles, and the expression of immune checkpoints, cancer cells suppress the cytotoxic properties of the immune system. The composition of the TME influences the clinical course and treatment response in cancer patients [78,79,80]. Over the years, researchers have demonstrated the significant role of bacteria in shaping the composition of the TME. Consequently, they can influence the functional status of the TME and affect immunosuppressive conditions.

**Figure 5:**
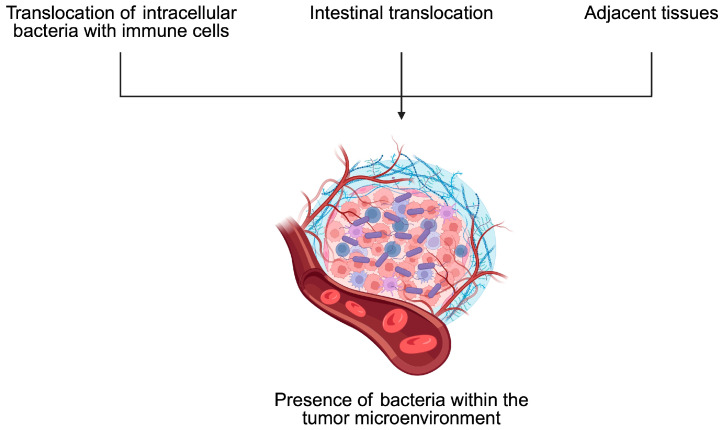
Bacteria can localize within tumors through several routes, such as intestinal translocation or through adjacent tissues. Furthermore, intracellular bacteria can travel within immune cells. Created in BioRender. Physiology, D. (2025) https://BioRender.com/a2lsxy3.

To begin with, cancer cell functionality can be affected by the activity of distant microbiomes, such as the one present in the intestine, as well as the composition of residual tumor bacteria. Regarding the latter phenomenon, the origin of microbiomes within the tumor is not entirely known. It is speculated that they could result from retrograde transport in the gastrointestinal system, changes in gut permeability, or transport through the adjacent tissue, while immunosuppressive conditions present within the tumor tissue could promote the growth of bacteria [81]. Moreover, intracellular bacteria can travel to the tumor tissues within immune cells [82] (Figure 5). Experiments on CRC and oral cancer demonstrated that bacteria tend to localize in less vascularized tumor niches. Moreover, these spots are characterized by a greater expression of immune checkpoints such as programmed cell death protein 1 (PD-1) and cytotoxic T-lymphocyte-associated protein 4 (CTLA-4) [83]. Importantly, intratumoral bacteria affect the clinical parameters of cancer patients and influence the composition of the TME. For example, in HCC tissue samples dominated by bacteria, greater infiltration of M2 macrophages was observed [81]. The M2 phenotype of macrophages is related to their immunosuppressive properties. Therefore, an enhancement of their presence promotes immunosuppressive conditions within the TME, thus driving cancer progression. In another study, researchers observed that CRC tumor tissues with the presence of bacteria were characterized by a greater presence of myeloid cells and lower abundance of CD4+ and CD8+ T cells compared to adjacent regions with no presence of bacteria [83].

The impact on the profile and functionality of the TME has been explored in greater detail in certain bacteria than in others. The important role of *H. pylori* in GC and MALT lymphoma has been previously mentioned. Recently, more light has been shed on the mechanisms linking this bacterium with the TME. Specifically, GC tumors positive for *H. pylori* demonstrated significant changes compared to tissue without the presence of bacteria. Analyses of immune density revealed that tumors with bacteria had a greater presence of PD-1 and PD-L1 cells. Moreover, a greater abundance of non-exhausted T cells and no significant difference regarding exhausted T cells were found. In clinical analysis, patients with tumors positive for *H. pylori* treated with immunotherapy demonstrated greater progression-free survival (PFS) than patients without a bacterial presence. Nevertheless, these findings corresponded to patients with microsatellite stable GCs. Those with microsatellite instability-high tumors demonstrated poorer immune-related PFS [84]. The impact of bacteria on the expression of PD-1, and thus on PFS, highlights an important aspect of the immunotherapy response based on the abundance of PD-1/PD-L1. GC is one of the malignancies for which the use of systemic immunotherapy is recommended, depending on the expression of PD-L1 [85]. Recently, it was confirmed that the addition of pembrolizumab to chemotherapy in first-line settings in patients with HER2-negative advanced GC provides clinical benefits in those with greater CPS scores [86].

**Figure 6:**
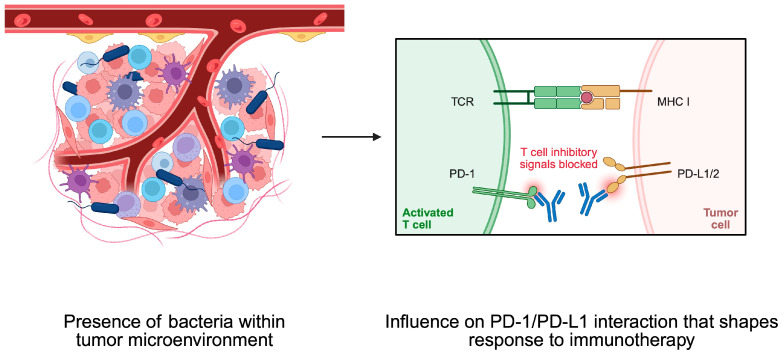
Bacteria within the tumor microenvironment shape its composition. They affect the PD-1/PD-L1 interaction, thus influencing the immunotherapy response. Created in BioRender. Physiology, D. (2025) https://BioRender.com/pht1cab.

Moreover, bacteria can affect the composition of the TME, which would limit the efficacy of immunotherapy. *Peptostreptococcus anaerobius* was found to enhance the presence of myeloid-derived suppressor cells (MDSCs) in the TME. MDSCs are immunosuppressive cells that contribute to the development of ICIs’ resistance. CRC mice models demonstrated a weaker response to anti-PD-1 treatment when *P. anaerobius* were injected into the tumor tissue [87] (Figure 6). Despite immunotherapy, it was recently observed that the composition of intratumoral bacteria can affect the radiotherapy response as well [88].

## 5. Bacteria-Mediated Cancer Therapy

### 5.1. Engineered Bacteria

Bacteria have emerged as a promising tool in cancer therapy due to their unique ability to home in tumors. The TME is characterized by a hypoxic, nutrient-rich, and immunosuppressive state, which fosters bacterial colonization [89,90,91,92]. The inherent chemotaxis and motility of bacteria allow them to actively migrate towards tumor sites. Therefore, bacteria could represent ideal candidates for targeted therapeutic interventions [91,92,93]. Early observations, including Coley’s use of bacterial toxins to activate the immune system, laid the foundation for modern bacterial therapies. In the contemporary era, significant strides in synthetic biology and genetic engineering have empowered researchers to harness bacteria, not only for their inherent therapeutic properties but also as precision drug delivery systems [90,91,94,95].

In recent years, there have been notable advancements in the field of cancer treatment, with bacteria being utilized in innovative ways. A notable strategy involves the direct reprogramming of the TME. For instance, genetically modified bacteria can be employed to deliver genetic cargoes to tumor-associated macrophages (TAMs), thereby inducing a shift in their polarization from the pro-tumor M2 phenotype to the antitumor M1 phenotype, thus potentiating immune-mediated tumor destruction [96]. Furthermore, bacteria are used as carriers to transport a variety of anticancer agents directly into tumor cells, including chemotherapeutic drugs, cytokines, siRNAs, and nanobodies [89,93]. This dual action, combining immune modulation with targeted drug delivery, has the potential to significantly improve therapeutic outcomes while reducing systemic toxicity [94].

**Figure 7:**
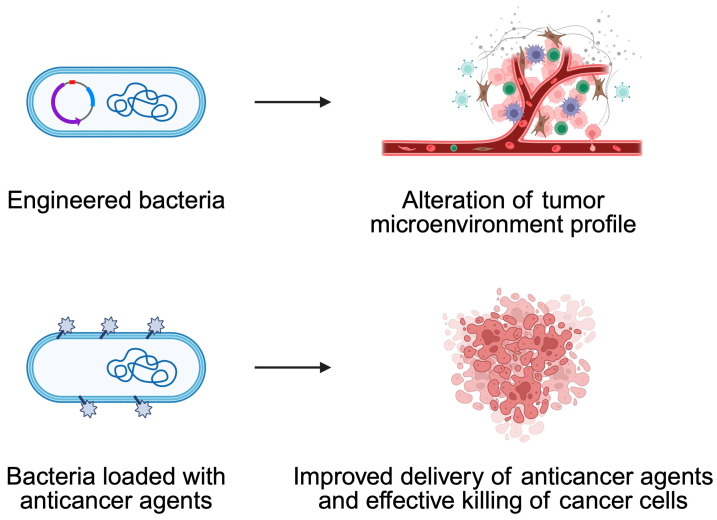
Bacteria can be engineered to alter the profile of the tumor microenvironment to increase pro-inflammatory properties or to improve immunotherapy efficacy. Furthermore, bacteria can be loaded with drugs to directly deliver them to cancer cells. Created in BioRender. Physiology, D. (2025) https://BioRender.com/qcnt7wx.

Engineered bacteria can be loaded with a broad range of anticancer agents [89,90,91,92,93,96]. Additionally, they can be programmed to synthesize and secrete therapeutic proteins, cytokines or enzymes that activate prodrugs directly within the tumor [90,92]. The release of these therapeutic agents is often regulated by genetic circuits, often based on quorum sensing or stimulus-responsive promoters, ensuring the precise and controlled delivery of drugs to the target site [91]. This approach has been shown to enhance local efficacy while minimizing systemic side effects [90,92,95,96]. The integration of bacterial carriers with nanomaterials has advanced the field considerably [91,92]. Chemical conjugation or encapsulation strategies enhance drug loading and targeting efficiency. For instance, when combined with nanoparticles, bacteria can enable multimodal therapies including chemotherapy, radiotherapy, and photothermal therapy, while also acting as immunoadjuvants to stimulate robust immune responses [89,92,93,95,96]. Recently, Shen et al. [97] described an interesting strategy to implement bacteria that can bind to the cells of nasopharyngeal cancer. Specifically, the authors showed that it is possible to use commensals to target cancer and release a chemotherapy prodrug which will activate in the presence of malignant cells. Consequently, this strategy should allow us to increase the efficacy of anticancer agent. The therapeutic showed promising results in an in vivo model of nasopharyngeal cancers (Figure 7).

Genetic engineering is a process that alters the nucleic acid sequence of organisms. This mechanism, aimed at generating new traits in genetically modified microorganisms (GEMs), involves the insertion or deletion of base pairs, as well as the introduction or inactivation of genes [98]. Bacteria are increasingly acknowledged as a key element of the TME, and genetic modifications of bacteria are prompting renewed interest in bacterial-based cancer therapies [99]. For example, researchers engineered bacteria producing outer membrane vesicles (OMVs) carrying therapeutic payloads, functioning as drug delivery carriers that directly target tumors, enhancing penetration, particularly in dense desmoplastic tumors in mouse models of breast and colon cancer. Hypervesiculating *E. coli* were designed to release OMVs at the tumor site, which were loaded with therapeutic peptides, including the enzyme Hyaluronidase (Hy) and the cytolytic protein Cytolysin A (ClyA), the latter of which degrades the extracellular matrix [100]. ClyA induces pore formation in cell membranes, disrupting ion balance and ultimately causing cell death [101].

An attenuated strain of *S. typhimurium* lacking the ability to synthesize guanosine 5′-diphosphate-3′-diphosphate (∆ppGpp; referred to as SAM) demonstrated improved tumor targeting [100]. The stimulation of regional tumor resistance and a high level of specificity have been demonstrated by this attenuated strain. Tumor cells, when induced by these mutated strains, can secrete antitumor cytokines such as IL-1β, IL-18, and TNF-α. At low concentrations, these cytokines may facilitate and promote tumor progression. Conversely, when produced at high concentrations, they have the potential to suppress tumor development [102]. Another study indicated that an attenuated strain of *S. typhimurium* (SAM-FC), engineered to secrete two therapeutic agents—ClyA and *Vibrio vulnificus* flagellin B (FlaB)—can function as an immunoadjuvant. This mechanism enhances the activation of tumor-specific T cell responses by increasing levels of damage-associated molecular patterns (DAMPs) and tumor-specific antigens (TSAs). Furthermore, while the expression of either ClyA or FlaB alone effectively eradicated tumors in respective mouse models (C57BL/6 and BALB/c), the combination of both agents resulted in a stronger and more durable antitumor response, effectively targeting both primary and metastatic tumors while also inducing long-term immunological memory [100]. A separate study indicated that attenuated *S. typhimurium* VNP20009, residing within macrophages, selectively released anti-PD-1 nanoantibodies upon reaching the tumor site. This study presents a novel macrophage-mediated strategy for the tumor-targeted delivery of VNP in a melanoma mouse model. By leveraging macrophage chemotaxis, controlled VNP release, and VNP’s tumor-colonizing properties, this approach enhances antitumor efficacy while minimizing acute organ injury. Treatment with VNP-loaded macrophages upregulated PD-L1 on cancer cells and downregulated PD-1 on tumor-infiltrating CD8+ T cells, potentially increasing tumor sensitivity to PD-1/PD-L1 blockade therapy.

### 5.2. How Can One Increase the Anticancer Effects of Bacteria?

Due to the fact that anticancer therapies are characterized by difficulties with appropriate selectivity and entry into the TME, bacteria can be programmed to avoid such obstacles. The effect of the bacteria themselves is too weak for tumors, which is why a number of strategies are used to help in this procedure.

Firstly, bacterial toxins, which are secreted in situ, lead to the direct destruction of cancer cells. This can also be achieved by expressing enzymes, which would affect the transformation of the non-toxic prodrug into cytotoxic drugs. Another mechanism uses the phenomenon of competition between bacteria and the appropriate factors that determine tumorigenesis. Processes such as angiogenesis and reduced sensitivity to apoptosis, but also avoidance of the immune system response, can be targeted due to the in situ delivery of polypeptides that exhibit pro-apoptotic activity (e.g., Fas ligand), cytokines (e.g., IL-2), and antiangiogenic factors [103]. In addition, external triggering was designed, which involves bacterial promoters characterized by a response to specific chemicals. This aims to release payloads under strictly defined conditions, i.e., the right dose selection at the right time. The main role of chemical indicators is to control the time of payload production and its release in the tumor area. Unfortunately, there is a fairly high risk of toxicity from this mechanism [104]. An example of application is the PBAD promoter, which is associated with ClyA (cytolysinA) and FlaB (flagellin) or with the initiation of cellular invasion by genetically modified *Salmonella* [105,106]. A better solution seems to be the use of physical signals that are not toxic or invasive. In addition, they could be used more precisely so that they can induce gene expression at a specific time. Optogenetics plays a very important role in this process. The mechanism of action is related to the sensitivity of proteins to light, which controls the corresponding genetic systems characteristic of specific cellular activities [107]. Due to the fact that visible light does not have the ability to penetrate into the deeper layers of tissue, a very important issue is the proper supply of photons. A system has been developed that converts near-infrared light into visible blue light [108]. This procedure was used in the modification of *E. coli* Nissle 1917, which introduced a gene circuit encoding an orthogonal heat switch. This strain was associated with a controlled ability to release TNFα or melanin, which elicited a significant antitumor response [109]. Another method used to express the expected protein with potential in cancer therapy is the use of bacterial promoters that can respond to quorum sensing (QS) [103]. Mechanisms involving QS will significantly facilitate the adaptation of the activity of bacteria that are found within the TME. In addition, the system can be programmed in such a way that there will be a time control depending mainly on the specific density of bacteria [110]. In addition, the system can be designed to be triggered by a response to multiple inputs, in order to prevent leaky expressions in response to a single signal. This was used for the expression of genes associated with the production of acyl-homoserine lactone (AHL) in *E. coli*. For this purpose, an AND-gate was used, and the mechanism can be activated in the presence of a signal from both QS and an exogenously introduced initiator [111].

Solid tumors are targeted by bacteria through distinct mechanisms. Therapeutic bacteria distribute to both tumor and healthy tissues upon systemic administration. *Salmonella* exhibits a preference for homing in on or persisting within the TME enriched with specific metabolites [112]. Passive and active mechanisms are utilized by bacteria to enter the bloodstream of tumor tissue. Initially, they may infiltrate the tumor due to passive entrapment within its disorganized vasculature, followed by further penetration driven by inflammation triggered by a sudden increase in TNFα levels in the tumor vessels [105]. Strategies to direct bacteria towards tumors using external magnetic fields are being developed to improve tumor colonization. Magnetic responsiveness can be achieved through the modification of commonly used strains, such as *E. coli* Nissle 1917 (EcN), with magnetic nanoparticles. *Magnetospirillum magneticum*, which naturally produce magnetic iron oxide nanocrystals, also represent an alternative approach for utilizing magnetotactic bacteria. The generation of torque occurs through the application of magnetic fields, causing the bacteria to perform a tumbling motion along blood vessels, which facilitates their passage through the endothelial barrier and enables them to reach tumors [113]. Chien et al. [114] described bacterial biosensors detecting oxygen, pH, and lactate, enabling preferential bacterial proliferation under physiological conditions. Controlled strains were developed by linking key gene expression to biosensor activity, ensuring selective growth. A study by Xiao et al. [115] described the integration of anaerobic bacteria with nanotechnology to develop bacteria–nanoparticle biohybrids for targeted chemotherapeutic delivery. *Bifidobacterium infantis* demonstrates the ability to selectively transport anticancer drugs to hypoxic tumor regions, thereby enhancing intratumoral drug accumulation while minimizing off-target distribution in healthy tissues. Nanoparticles (NPs) can increase a drug’s solubility and biocompatibility, as well as prolong its circulation time in the blood. Improved drug delivery to the tumor and a subsequent better treatment outcome occur through a modification of the NP surface. The properties of *B. infantis* in terms of delivering adriamycin nanoparticles (DOX-NPs) to hypoxic solid tumors were recently examined. To obtain the Bif@DOX-NP biohybrids, DOX was encapsulated within bovine serum albumin (BSA) and then incubated with a *B. infantis* suspension. Due to the hypoxia-targeting feature of the bacteria, the hybrids accumulated within the tumors. Table 1 summarizes bacterial properties that could be utilized in oncology, while Table 2 demonstrates selected the anticancer properties of engineered bacteria strains.

## 6. The Potential of Probiotics in Cancer Treatment

The strong involvement of bacteria in shaping the response to immunotherapy warrants investigations regarding the potential use of probiotics as an adjuvant treatment. In vitro experiments have demonstrated that probiotics possess beneficial properties in modulating cancer cell proliferation and apoptosis [116]. The significant antiproliferative effects and/or induction of apoptosis in musculus colon carcinoma (HGC-27), as well as human colon cancer cell lines (Caco-2, DLD-1, HT-29), has been highlighted by several researchers [117,118,119,120,121]. Probiotic bacteria are also recommended to enhance the production of SCFAs in the intestine. Their role is to improve the integrity of the intestinal epithelium; reduce bacterial translocation; regulate epithelial cell proliferation and differentiation; enhance nutrient absorption; serve as energy substrates for the liver, skeletal muscles, heart, and brain; prevent hyperinsulinemia; and exert anti-inflammatory effects [122]. SCFAs positively impact human health by serving as the primary energy source for colonocytes and demonstrating antimicrobial and anti-inflammatory properties. Butyrate, a prominent SCFA metabolite, is considered crucial in the prevention and treatment of CRC as it enhances colon cell function through promoting cell cycle arrest, induces the apoptosis of cancer cells, and stimulates immunomodulation [123]. *Lactobacillus reuteri* is a Gram-positive, facultatively anaerobic bacteria, which naturally inhabits the intestines of almost all vertebrates and mammals [124]. Treatment with *L. reuteri* significantly suppresses pancreatic ductal adenocarcinoma tumor growth and increases NK cell infiltration into the TME. The depletion of NK cells alleviated the antitumor effects of *L. reuteri*, which demonstrates the important relationship between *L. reuteri* and anticancer response mediated by NK cells. A clinical analysis of pancreatic cancer patients showed that a greater presence of fecal *Lactobacillus* correlated with prolonged PFS and OS [125]. Another study showed that the oral administration of *Lactobacillus casei* elicited a strong Th1 immune response and promoted cytotoxic T cell infiltration within the tumor tissue of colon cancer animal models [126]. A probiotic mixture consisting of *B. longum, B. bifidum, L. acidophilus,* and *L. plantarum* enhanced CD8⁺ T cell activation and their presence in tumor tissue. The mixture suppressed mouse colon cancer CT26 tumor growth [127]. Another study designed a probiotic microgel delivery system (L. reuteri@(SA-CS)₂) using layer-by-layer encapsulation with alginate and chitosan to modulate gut microbiota dysbiosis and enhance antitumor therapeutic efficacy. SCFAs produced by *L. reuteri* can induce apoptosis in certain tumor cells, modulate the gut microbiota, reduce harmful bacteria, and serve as nutrients for other beneficial microbes, particularly butyrate-producing probiotics [128].

Importantly, it should be highlighted that there are controversial results regarding the role of probiotics in improving anticancer treatment in clinical settings. Partially, contrary results could result from difficulties in translating preclinical success to clinical benefit and, therefore, introducing them to clinical practice. For instance, the use of probiotics was not related to significant changes in overall survival (OS) or PFS in the cohort of patients with advanced small-cell lung cancer treated with chemo-immunotherapy [129]. Nevertheless, the population that administered probiotics was small. Moreover, accumulating preclinical studies shed light on the potential use of probiotics, which could warrant the conduction of future clinical trials. Mechanistically, probiotic bacteria produce metabolites that can affect the composition of the TME and influence the response to treatment. For instance, butyrate-producing bacteria were found to promote pro-inflammatory changes in cancer TMEs, thus enhancing the benefits of immunotherapy in vivo [130,131]. 

## 7. Conclusions

To conclude, bacteria and cancer are strongly interconnected. Alterations in the microbiome and the direct influence of bacteria within the TME can shape the carcinogenesis and treatment response. Current evidence highlights the potential of analyzing the microbiome to analyze cancer properties or even the risk of developing cancer. Due to specific immunosuppressive conditions present within the TME, bacteria can localize in tumor niches. Importantly, these bacteria shape the composition and function of TME, thus affecting tumor growth and treatment resistance. Immunotherapy has now entered daily clinical practice. It is known that the composition of the TME affects immunotherapy response. As intratumoral bacteria can shape the profile of cells within the TME, they can indirectly contribute to treatment success or immunotherapy resistance. As certain bacteria can accumulate within the TME and bind to cancer cells, these properties could be used to design a novel class of anticancer agents with better delivery precision and efficacy.

Surgical resection or the biopsy of the tumor could perhaps be examined microbiologically, and the results could be used to determine the type of systemic treatment in the future. Analyses of both the TME and bacterial abundance could provide a significant amount of information that will shape further treatment.

## Figures and Tables

**Table 1:** The properties of bacteria that could play a beneficial role in oncology.

Characteristic	Description	References
Tumor Targeting	Bacteria such as *Escherichia coli*, *Salmonella*, and *Listeria monocytogenes* naturally migrate toward and colonize the hypoxic and necrotic regions of tumors due to their chemotactic abilities and motility.	[92]
Adaptability to the TME	The unique conditions within the tumor microenvironment—such as low oxygen levels, acidity, and abundant nutrients—create a favorable niche for bacterial survival and proliferation, which is not present in healthy tissues.	[92]
Immune Activation	Certain bacteria can stimulate both innate and adaptive immune responses, acting as natural adjuvants to enhance antitumor immunity.	[90]
Genetic Manipulability	Advances in genetic engineering have enabled the modification of bacterial strains to attenuate virulence, control therapeutic gene expression, and incorporate regulatory circuits, thereby increasing safety and efficacy.	[90,91]

**Table 2:** Anticancer properties of selected engineered bacterial strains.

Bacterial Strain	Key Properties	References
*Escherichia coli* (e.g., BL21, DH5α)	Genetically engineered to produce therapeutic agents; effectively colonizes tumor tissues due to natural tumor-targeting abilities.	[90,92]
Salmonella typhimurium	Exhibits robust motility and chemotaxis; engineered into attenuated “suicide” strains that deliver cytotoxic proteins and prodrug-converting enzymes to induce apoptosis.	[90,94]
*Listeria monocytogenes*	Stimulates the immune system; induces direct tumor cell death through mechanisms involving reactive oxygen species (ROS) and mitochondrial disruption.	[94]
Probiotic Strains (*Lactobacillus plantarum*, *Enterococcus faecalis*, *Bifidobacterium* spp.)	Exhibit selective anticancer activity; investigated for their potential to inhibit tumor growth and work synergistically with conventional therapies.	[95]

## Data Availability

Not applicable.

## Abbreviations

The following abbreviations are used in this manuscript:

SCFAs	Short-chain fatty acids
GPCRs	G-protein-coupled receptors
Tregs	Regulatory T cells
TGF-β	Transforming growth factor-β
DCs	Dendritic cells
TME	Tumor microenvironment
APCs	Antigen-presenting cells
ROS	Reactive oxygen species
PI3K	Phosphatidylinositol-3-kinase
TLR4	Toll-like receptor 4
NF-κB	Nuclear factor-kappa B
GC	Gastric cancer
cagA	Cytotoxic-associated gene
vacA	Vacuolating cytotoxin A
oipA	Outer inflammatory protein
CRC	Colorectal cancer
HCC	Hepatocellular carcinoma
PD-1	Programmed cell death protein 1
CTLA-4	Cytotoxic T-lymphocyte-associated protein 4
ICIs	Immune checkpoint inhibitors
PFS	Progression-free survival
OS	Overall survival
TAMs	Tumor-associated macrophages
OMVs	Outer membrane vesicles
NPs	Nanoparticles
